# Non-Invasive Characterisation of Bromoil Prints by External Reflection FTIR Spectroscopy

**DOI:** 10.3390/molecules29245833

**Published:** 2024-12-11

**Authors:** Raquel Freixas-Jambert, Cristina Ruiz-Recasens, Alejandra Nieto-Villena, Marta Oriola-Folch

**Affiliations:** 1 Research Group of Conservation of Cultural Heritage, Art and Conservation Department, Fine Arts Faculty, University of Barcelona, Pau Gargallo, 4, 08028 Barcelona, Spain; 2 Department of Art History, Faculty of Geography and History, University of Valencia, Avda. de Blasco Ibáñez, 28, 46010 Valencia, Spain

**Keywords:** bromoil process, photography, oil positives/prints, FTIR spectroscopy, barite layer, pigment process, gelatine, greasy ink, reflection mode

## Abstract

The bromoil process, developed in 1907, was a photographic technique highly esteemed by pictorialist photographers for its capacity for image manipulation, which aligns its expressiveness with traditional pictorial techniques. Despite the artistic and technical value of bromoils and their prevalence in renowned collections, there is little research on their composition and structural characteristics. This study uses non-invasive external reflection FTIR spectroscopy to characterise 16 bromoil prints dating from the 1920s to 2010. FTIR spectroscopy allowed the discrimination of key components such as cellulose, gelatine, pigments, and oils, elucidating the structural and compositional complexity of bromoil prints. The study presents new perspectives on the traditionally recognised structure of bromoil prints, based on FTIR spectroscopy results along with evidence from microscopic examination, particularly regarding the role of certain strata in their identification. These results suggest a need to reconsider the understanding and characterisation of bromoil prints in relation to the currently available literature. This research also proposes a measurement model adapted to the studied samples and addresses the advantages and limitations found in the different types of equipment used. It thus proposes a key methodology for the identification and provides a source for the physicochemical studies of photographic processes

## 1. Introduction

Bromoil is one of the so-called photographic pigment processes. It was first developed in 1907 as a refinement of Rawlins’s oil process (invented in 1904), and it represents the culmination of decades of research and experimentation aimed at identifying a definitive noble process. The bromoil process was particularly embraced by pictorialist photographers due to its high capacity for manipulation and intervention during the processing of the image, as well as its unique visual characteristics and proximity to the pictorial aesthetic of the images it produced [1,2,3,4,5]. 

The final image, although photographic, is materialised from a greasy ink similar to the one used in lithography. It is formed on a silver gelatine developing-out paper, usually bromide paper, from which the metallic silver has been removed by bathing the paper in different chemical solutions during the production of the bromoil. This reaction hardens the gelatine and renders it hydrophobic to a degree that is proportional to the quantity of silver present in the image. This results in the formation of a gelatine matrix that can be moistened and inked in accordance with its degree of hardening. The outcome is a pigment image comprising greasy ink that faithfully reproduces the original silver image. Furthermore, the author has the option of modifying and adding changes to the image during the inking process and subsequent retouching with remarkable ease, a capability that is unique to this historical photographic process.

Due to the intricate nature of the production process and the singularity of the resulting object, bromoil prints are often considered to possess remarkable artistic and technical value. They are frequently included in a multitude of private and public collections, held within archives, museums, and cultural institutions. Nevertheless, there is a notable absence of scientific studies and research focused on these photographic processes.

To gain a comprehensive understanding of the composition and technical aspects of oil processes, it is essential to seek out primary sources from the era of the greatest production of these photographs, which spans the first decades of the 20th century [6,7]. Despite the aforementioned references describing the materials utilised in these processes, it remains unclear which compounds constitute these photographs, a factor of significant importance for their conservation. Potential variations between authors, residual substances not eliminated by the baths, by-products generated during the manufacturing process, and alterations due to the passage of time, all contribute to this uncertainty. Secondary sources of information on these processes are also relatively scarce and mainly focus on the production aspects. Moreover, most of them are compilations of data from primary sources.

A review of the literature on the characterisation of historical photographic processes through morphological study reveals a notable scarcity of references to oil processes. Among the sources consulted, the Graphics Atlas [8] stands out as the most comprehensive on this topic. Together with Pavão’s manual [9], these constitute the sole direct sources on the morphological identification of bromoil prints. Moreover, the limitations of morphological characterisation preclude the determination of composition and deterioration processes, as well as the distinction between photographic processes that look similar and are easily mistaken for one another, such as silver gelatine DOP or dichromate gum and its derivatives [8].

It is therefore necessary to develop a more objective research methodology that relies on advanced identification techniques in order to obtain detailed and specific data on the composition and interaction between materials and their environment [10]. FTIR spectroscopy has yielded optimal results in the characterisation of photographic materials. Some studies have made significant advances in the possibilities of studying photographs and paintings by using external reflection FTIR spectroscopy (ER-FTIR), a non-destructive and non-invasive method [11,12]. However, the majority of existing FTIR studies focus on silver processes, with only a few examining alternative processes [10,13,14,15,16,17,18,19]. A recent study has applied instrumental analysis techniques to the study of bromoil prints [20]. Nevertheless, it does not address the numerous inconsistencies identified in the existing literature on the structure and composition of bromoil prints, which remain unresolved.

For example, some authors include bromoil positives in the category of two-layer photographic processes [9], thereby indicating that these do not contain the barite layer that is typical of photographic papers. However, other reference sources [8] explicitly state that the barite layer is a key in the identification of bromoil prints.

In light of the aforementioned considerations, this study seeks to examine the potential of FTIR spectroscopy in external reflection mode as a non-destructive characterisation tool for bromoil prints. The applicability of this technique offers significant results in the analysis of cultural and artistic heritage [21], and it offers some important advantages such as the portability of some equipment [22,23], the ability to explore both organic and inorganic compounds [24,25], the possibility of discerning between the different layers of a sample [11,26], and the capacity to obtain valuable information without damaging the sample [14,27]. This approach allows us to expand our knowledge of the composition and structure of bromoil prints and to determine the information that can be obtained by reflection FTIR spectroscopy for their characterisation. In order to optimise the use of FTIR spectroscopy and obtain valuable results for the photographic process under study, the aim is to describe the methods and define the parameters to be used, as well as to evaluate the advantages and limitations offered by both desktop and portable spectrometers.

## 2. Results and Discussion

### 2.1. Results

The results demonstrate that the FTIR spectroscopy in external reflection mode is a valuable technique for the study and characterisation of bromoil prints. The methodology employed to carry out the analysis, along with the selection of the areas to be analysed based on different image densities, has provided information about the materials present in the works and their stratigraphic position. This allowed for the characterisation of the layered structure of the studied print, based on the spectral response of the materials and the location of the measurement points.

FTIR analysis has made it possible to identify the main compounds present in each of the layers that make up the bromoil prints: cellulose, as the main component of the paper support; the barite layer, when this is present in the work immediately above the paper; the gelatine, which is present in the next layer; and, in the most superficial layer, the oils and certain pigments of the image-forming layer.

Each of these compounds has been detected with a greater or lesser degree in the resulting FTIR spectrum, depending on the proportion in which it is present at the point analysed and/or on the presence or absence of other upper layers, which may make its presence more evident or, on the contrary, make its identification more difficult.

In accordance with the above, after this study, we can affirm that, in bromoil prints, the intensity of the FTIR response of the surface layer of the greasy ink is directly proportional to the density of the image, while it is inversely proportional in the case of gelatine, barite, and cellulose. Taking this aspect into account, the comparative study of the zones of different density (Dmax, Dmid, and Dmin) allows us to characterise the stratigraphic structure of the copy.

#### 2.1.1. Cellulose

**Figure 1:**
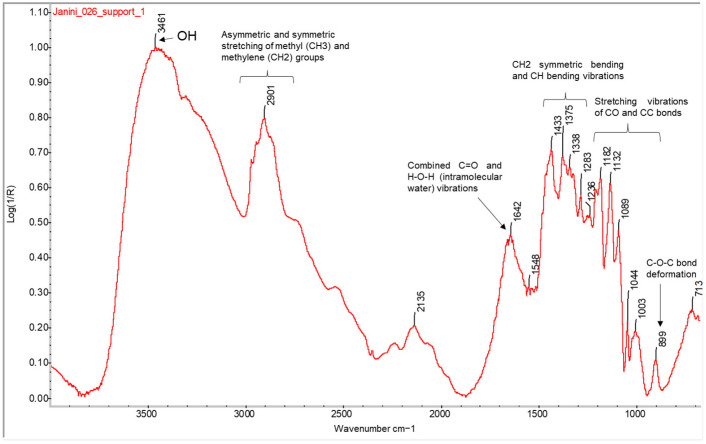
FTIR spectrum in external reflection mode of the reverse side of the primary support of the *Janini_026* bromoil sample obtained with the Nicolet™ iN10 MX spectrometer (ThermoFisher Scientific, Waltham, MA, USA) of the Scientific and Thechnological Centers at the University of Barcelona (CCiTUB) (Barcelona, Spain). A clear spectrum of cellulose is identified from the assignment of the main bands.

The first layer of this structure is the paper support. The analysis of the back of the bromoil samples allows us to characterise it without interference from the other layers superimposed on the front to form the photographic print. The backs of all the bromoil samples selected in the study gave a similar response in all cases, corresponding to the spectral characteristics of cellulose, the main component of the paper, and with a high response in FTIR. For example, in Figure 1, which shows the spectrum obtained from the analysis of the back of the *Janini_026* bromoil print, the broad and strong band of the OH group at 3460 cm^−1^, and the absorptions in the region between 2970 and 2860 cm^−1^ approx., which presents a broad band centred at 2901 cm^−1^, are related to the asymmetric and symmetric stretching of the methyl (CH_3_) and methylene (CH_2_) groups [28,29]. The bands located at 1433, 1375, 1338, and 1323 cm^−1^ are assigned to CH_2_ symmetric bending and CH bending vibrations [11,29,30]. A band appears at 1642 cm^−1^, corresponding to the combined C=O and H-O-H (intramolecular water) vibrations [28]. Finally, the region from 1180 to 1040 cm^−1^ corresponds to the stretching vibrations of the CO and CC bonds in the cellulose chain [11]. The small peak appearing near 1000 cm^−1^ could also be attributed to CO stretching vibrations, while the absorption peak observed at 899 cm^−1^ appears to correspond to the C-O-C bond deformation [11,31], which is assigned to the amorphous region in cellulose [29]. 

**Figure 2:**
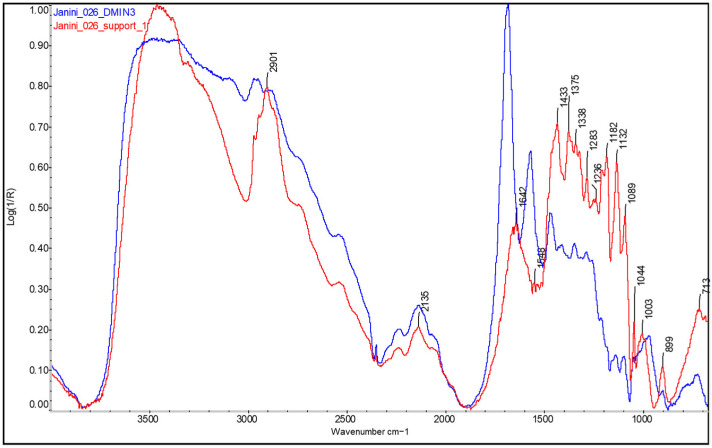
FTIR spectrum of the Dmin (see blue line) and paper support of the bromoil sample *Janini_026*, obtained using the Nicolet™ iN10 MX desktop spectrometer (ThermoFisher Scientific, Waltham, MA, USA) at the CCiTUB (Barcelona, Spain). The bands of the protein material (binder) cover a major part of the cellulose spectral signature. Only the absorptions below 1130–700 cm^−1^ could be faintly intuited, although their interpretation is doubtful.

From the front, the cellulose is hardly visible at most points in the Dmin and edge regions due to the strong response of the proteinaceous material forming the photographic binder layer on the paper support. A large part of the spectral bands of the cellulose are covered by the amide bands of the protein binder (~1690–1470 cm^−1^) at the fingerprint region (see Figure 2, blue spectral line). Only the absorptions below 1130–700 cm^−1^ could be faintly intuited, although their interpretation is doubtful, and this information is not sufficient to identify or corroborate the presence of cellulose. Furthermore, it is also not possible to distinguish the bands of the OH, methyl (CH3), and methylene (CH2) groups of cellulose located in the upper range of the spectrum (approximately 3400–2860 cm^−1^) due to the spectral distortions that appear in the bands of this range. Nevertheless, the objective is not to corroborate the presence of cellulose in the sample, as this is plausible through general observation of the sample and demonstrable by analysing the back of the copies (see Figure 1), but rather to study how the upper layers, such as the protein binder layer, affect its detection from the front, even in areas of minimal image density. Therefore, it confirms that there are indeed other layers located between the paper support and the material forming the image (corresponding to the Dmid and Dmax areas). These data are very useful when characterising photographic prints because, when correlated with observations from microscopic study, they allow differentiation between photographic copies with one and those with two or three layers (the former without photographic binder) and, thus, between the different photographic processes that characterise them. Moreover, as discussed in the results discussion, the detection of protein material covering the absorptions of cellulose in the Dmin areas is an indication that discards processes like gum bichromate or carbon.

#### 2.1.2. Gelatine

**Figure 3:**
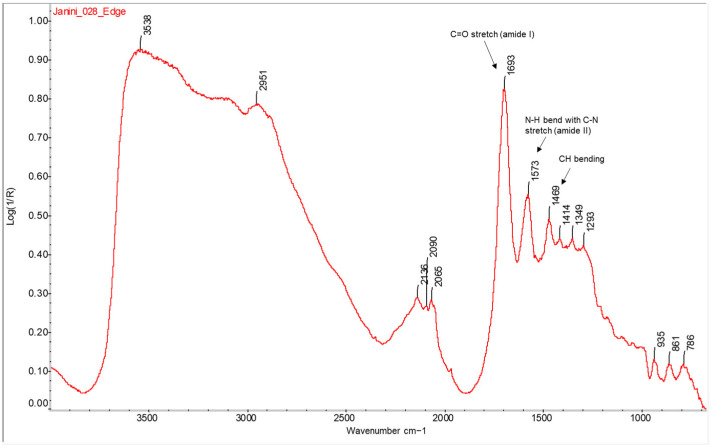
FTIR spectrum in the reflection mode of the area corresponding to the non-image edge (absence of ink) of the bromoil sample *Janini_028*. The bands of amides I, II, and III are clearly visible in the region ~1690–1460 cm^−1^ corresponding to the protein material. Spectrum recorded on a Nicolet™ iN10 MX desktop spectrometer (ThermoFisher Scientific, Waltham, MA, USA).

Measurements in the areas corresponding to the non-image margins and the areas of minimum image density allow the IR signals of the protein binder to be detected with a minimum of spectral interference from other materials present in the samples, particularly the oil-based ink in the image. The spectra obtained in these areas are quite sharp and show intense bands due to the fact that the binder layer is the first surface layer in these areas of the bromoil prints (regardless of any loose and dotted pigment particles). In Figure 3, the two sharp and intense bands of amide I (carbonyl absorption) and II (combination of C-N and N-H vibrations) at ~1693 and ~1573 cm^−1^, respectively, are clearly distinguishable [28,32]. The CH bending vibration occurring in this spectrum near 1469 cm^−1^ is also clearly visible [32]. The bands corresponding to amides A and B, which usually appear between 3300 and 3100–3030 cm^−1^ [33], are not well distinguishable due to the distortion in this range.

**Figure 4:**
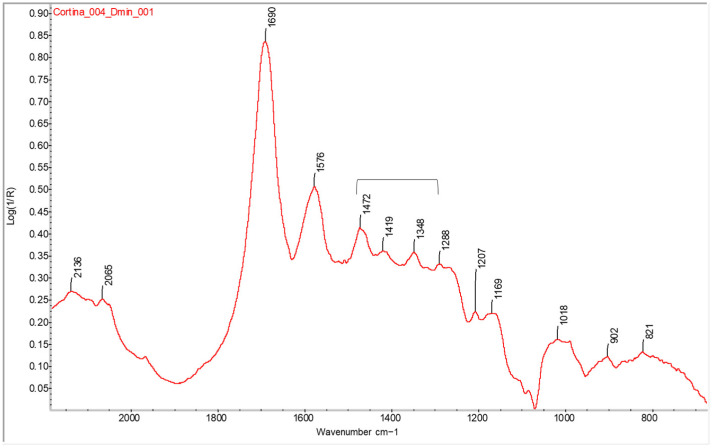
Detail of the FTIR spectrum in the reflection mode of the Dmin region of a photograph of a bromoil print (*Cortina_004*), showing the typical pattern of the gelatine photographic binder. The spectrum was obtained using a Nicolet™ iN10 MX desktop spectrometer (ThermoFisher Scientific, Waltham, MA, USA).

The results demonstrate the effectiveness of FTIR spectroscopy in identifying the proteinaceous binder in bromoil prints but also the possibility of distinguishing it from albumen, which is not present in these works, although it is present in prints made using other photographic processes. Figure 4 clearly shows the three specific bands of gelatine located at approximately 1472, 1419, and 1348 cm^−1^, which gradually decrease in intensity, with the first being much more noticeable, but all three show a significant response (even with interference from the spectral bands of other compounds such as cellulose), unlike albumen, which has bands located at 1451 and 1399 cm^−1^ of very similar intensity [10].

**Figure 5:**
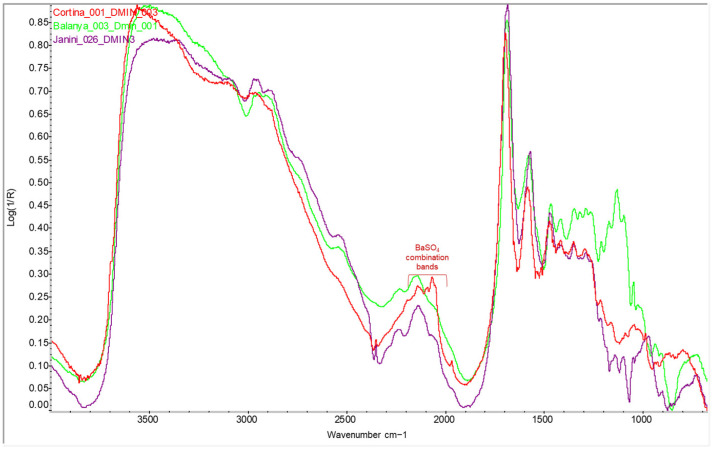
FTIR reflectance spectra taken through the Dmin areas of the bromoil prints *Janini_026* (first half of the 20th century) (purple), *Cortina_001* (1940s—late 1950s) (red), and *Balanya_003* (2009–2010) (green). Despite the difference in the relative intensities of the spectral bands, which could not be related to the age of the print, there is no significant difference in the bands of the protein material between the three prints. In the sample *Cortina_001*, it is also possible to distinguish the combination bands of barium sulphate (red). Spectra acquired using the Nicolet™ iN10 MX desktop spectrometer (ThermoFisher Scientific, Waltham, MA, USA).

In this study, the spectral interpretation of the photographic binders of the copies did not reveal any relevant differences that would indicate a change in the period or authorship of the bromoil prints analysed, not even between the oldest (first decades of the 20th century) and the most recent (2009–2010) (see Figure 5). It should be noted, however, that although the bromoil samples analysed come from three different collections, each with specific storage conditions, no obvious conservation problems or samples in an advanced state of degradation were found. The only plausible difference is the appearance of combination bands related to barium sulphate from the barite layer in the sample *Cortina_001*, located beneath the gelatine layer (see Figure 5, red spectral line).

As mentioned above, the protein binder in the bromoil samples is detected by FTIR in a way that is inversely proportional to the image density, confirming that this analytical technique can be used to characterise the material as coming from a layer below the image, i.e., the photographic support. This means that areas of lower image density (Dmin) show a higher intensity of gelatine bands, while, in areas of maximum density (Dmax), these bands are significantly reduced. This is an important point, as it allows bromoil positives to be distinguished from other very similar photographic positives, such as DOP (chemical development) prints or carbon prints. In DOP positives, the gelatine binder is detected with the same intensity over the entire surface, as it is the most superficial layer of the copy (except for possible coatings), since it contains the silver particles of the image inside (emulsion) [10]. In carbon prints, on the other hand, the pattern is the opposite; the amide bands I and II of the gelatine are more clearly detected in the areas of maximum density of the image, where there is a greater thickness of pigmented gelatine [34].

**Figure 6:**
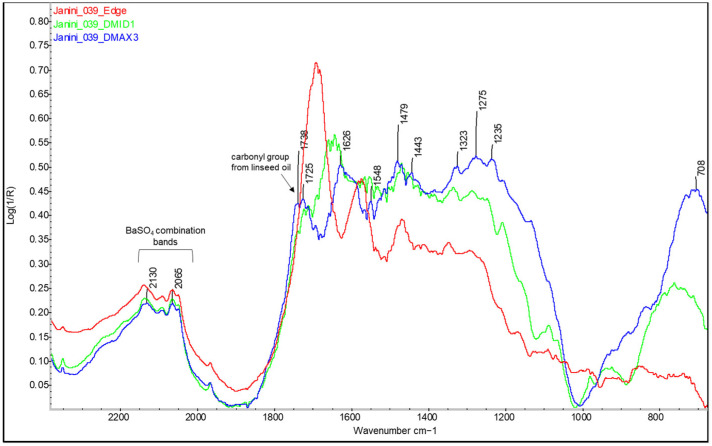
Comparison of the FTIR spectra corresponding to the non-inked edge (red) in the Dmid (green) and Dmax (blue) regions of the bromoil print *Janini_039*. As the image density increases, and with it the concentration of oil-based ink, the amide bands of the protein lose definition and become obscured by the overlapping of the typical bands of oils in this range of the spectrum. In the spectrum, it is also possible to observe the combination bands of barium sulphate from the barite layer. Spectra obtained using a Nicolet™ iN10 MX desktop spectrometer (ThermoFisher Scientific, Waltham, MA, USA).

**Figure 7:**
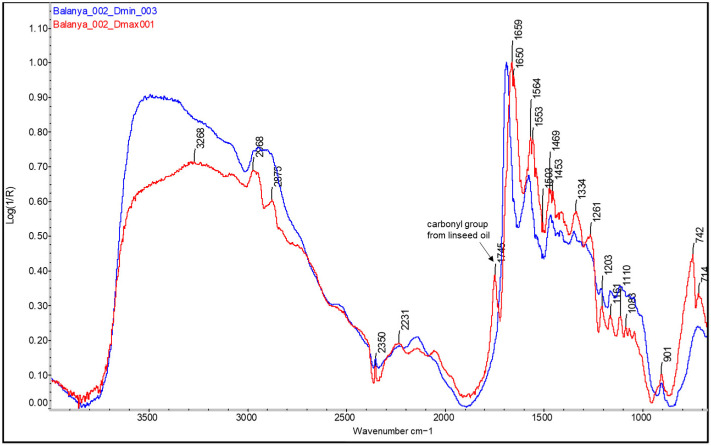
FTIR spectra of the Dmin (blue) and Dmax (red) regions of the bromoil sample *Balanya_002*, showing the loss of definition of the binder bands in the Dmax region of the image. The spectra were acquired using a Nicolet™ iN10 MX desktop spectrometer (ThermoFisher Scientific, Waltham, MA, USA).

**Figure 8:**
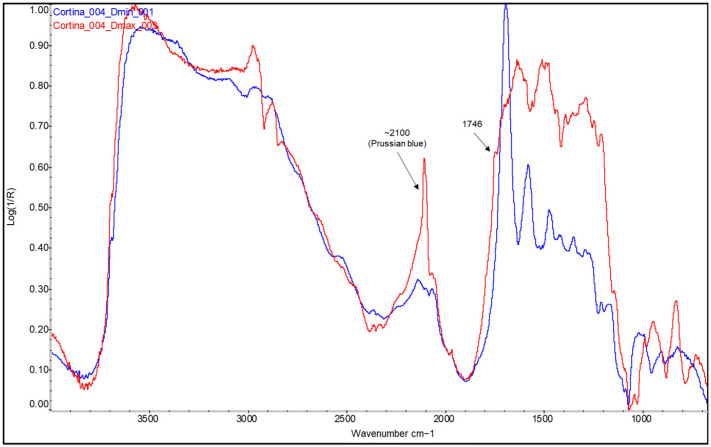
FTIR spectra of the Dmin (blue) and Dmax (red) regions of the bromoil sample *Cortina_004*. The amide bands of the protein are barely recognisable in the spectrum obtained in the Dmax area. Along with the appearance of the shoulder at 1746 cm^−1^ related to the linseed oil present in the ink, the characteristic band of the triple CN bond of Prussian blue also appears at Dmax. The spectra were acquired using a Nicolet™ iN10 MX desktop spectrometer (ThermoFisher Scientific, Waltham, MA, USA).

For example, looking at Figure 6, broad shoulders between 1715 and 1745 cm^−1^ begin to form in the areas of medium density (Dmid) as the presence of ink, and therefore, the drying oil increases. When the ink concentration is at its maximum exponent, this back is defined as a broad band or the merging of two bands, at ~1715 cm^−1^ and ~1745 cm^−1^, in the areas of maximum density (Dmax) (see Figure 6, Figure 7 and Figure 8). In parallel with the appearance of these bands related to the linseed oil present in the ink, specifically the carbonyl group band at 1750–1740 cm^−1^ [32] and a small peak at around 1715 cm^−1^ related to the level of polymerisation of the oil and suggesting the formation of acidic carboxylic products [35], there is a decrease in the sharpness and shape of the bands related to the protein material and, in some cases, also in their relative intensity, around 1690, 1572, and 1460–1470 cm^−1^ (see Figure 6, Figure 7 and Figure 8). Along with the appearance of the shoulder at 1746 cm^−1^ related to the linseed oil present in the ink, the characteristic band of the triple CN bond of Prussian blue also appears at Dmax in the sample *Cortina_004* (see Figure 8, red spectral line).

It is therefore shown that this behaviour is directly related to the presence of the surface layer of greasy ink that forms the image of the bromoil print. In other words, it is mainly associated with the interference of a layer on top, the oily ink, which is interposed between the gelatine substrate and the light beam of the spectrometer, so that the response of the binder is less strong, affecting, in some cases, the relative intensity and sharpness of the protein bands. We can therefore confirm that the variability in the relative intensities and definition of the bands gives us information about the stratigraphic position of the different layers detected in the FTIR spectrum.

#### 2.1.3. Barite

**Figure 9:**
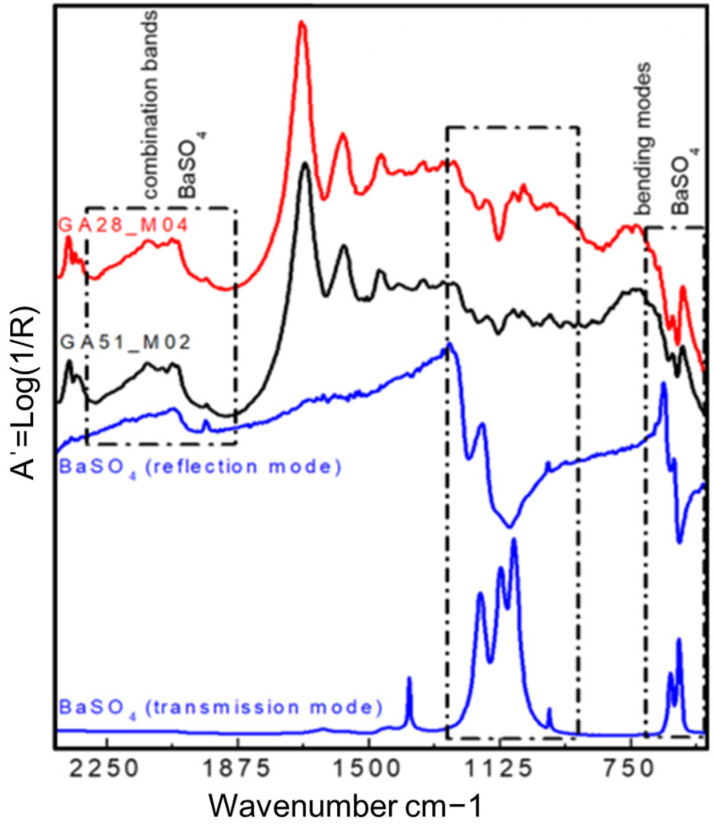
Comparing transmission mode and reflection mode spectra recorded from a barium sulphate reference compound—blue spectral lines—with the spectra obtained in the areas of minimum density (Dmin) of two original bromoil samples from Pla Janini (*Janini_028* and *Janini_051*). The characteristic IR bands of barium sulphate and their corresponding assignments are highlighted by black boxes in the range of 2250 to 1870 cm^−1^ (combination bands) and the v_4_ bending vibration of the SO2^−4^ between 700 and 550 cm^−1^. Spectra obtained with the Alpha mobile spectrometer (Bruker Corporation, Billerica, MA, USA).

In contrast to the spectral overlap generated between the protein bands, which hide the IR signals of the cellulose of the paper layer, the results show that the presence of barium sulphate, the main component of the barite layer, does not generate any significant interference for the detection of the other layers of the photographic support (gelatine and cellulose) (see Figure 9).

In fact, the identification of barite in bromoil prints by FTIR presents certain difficulties, resulting from the superposition of the stretching modes located in the range of 1280 to 970 cm^−1^ (indicator generally used for the detection of the sulphate anion) with bands of different materials present in the sample, such as cellulose, gelatine, and, in some cases, silicate compounds such as kaolinite. The second problem, as stated by Miliani et al. [24], is that the bands located between 1200 and 1000 cm^−1^, typical of the asymmetric stretching ν_3_ of the tetrahedral anion SO_4_^2−^ present in barite, are not observed in the reflection mode due to its high absorption index k, by which all the ν_3_ bands of the sulphate compounds are affected by the so-called Reststrahlen effect. As a result, they appear in the spectrum as inverted bands [24], which are difficult to recognise when they are due to the different materials that make up bromoil prints and which can sometimes cause confusion when the experimental spectra are interpreted, especially if no comparison with another spectrometer or technique (as transmission mode) is available [36].

However, in the bromoils analysed with the Alpha mobile spectrometer (Bruker Corporation, Billerica, MA, USA), the combination bands in the range of 2250 to 1870 cm^−1^, together with the v_4_ bending vibration of the SO_2_^−4^ between 700 and 550 cm^−1^, can be read as an indication of barium sulphate, especially when clearly detected in the areas of minimum density or at the margins without images of the combination bands (see Figure 9).

**Figure 10:**
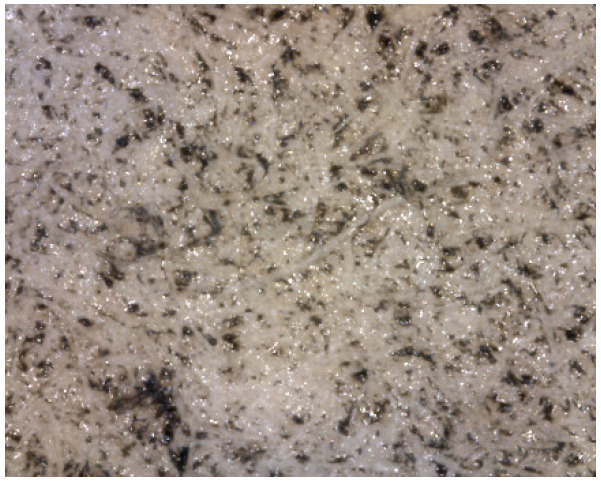
Janini_026_Dmin_1 (5) 180× (Digital Surface Microscope).

**Figure 11:**
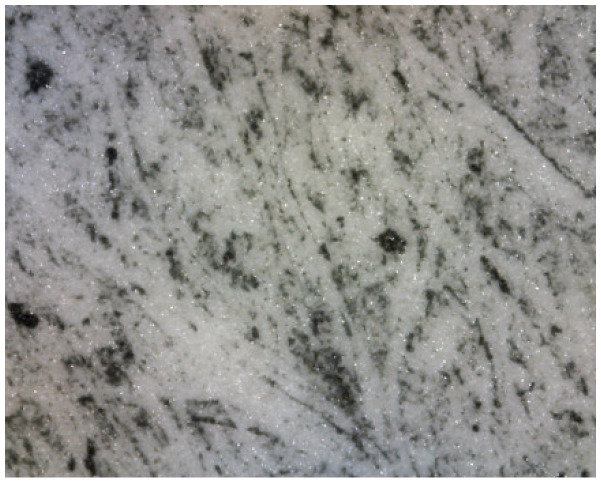
Janini_028_Dmin_2 (2) 180× (Digital Surface Microscope).

**Figure 12:**
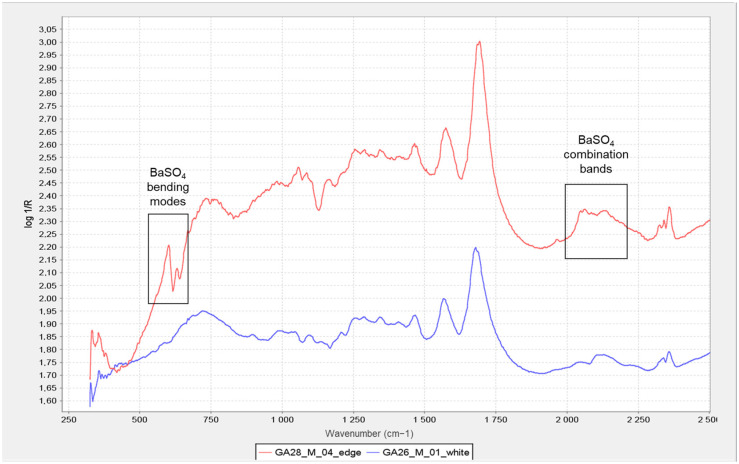
A comparison of two-point FTIR spectra corresponding to the Dmin region of a barite-coated bromoil sample, *Janini_028* (red), and a barite-free sample, *Janini_026* (blue). The combination bands of barium sulphate are visible in the 2500–1870 cm^−1^ region in the *Janini_028* sample (red), as are the two strong inverted bands at 640 and 617 cm^−1^ corresponding to the bending modes; neither of these bands is visible in the *Janini_026* sample (blue). Spectra obtained with the Alpha mobile spectrometer (Bruker Corporation, Billerica, MA, USA).

This information was very relevant in confirming the presence of the barite layer in some of the samples where the previous microscopic examination had not given clear results (see Figure 10 and Figure 11), especially in the case of very thin barite layers. For example, in the case of samples *Janini_028* and *Janini_026*, the analysis with the mobile FTIR spectrometer has allowed us to confirm the presence or absence of the barite layer, information that was intuitively present in these samples at the morphological level (see Figure 10 and Figure 11). On the one hand, the measurements carried out in the Dmin regions of sample *Janini_028* (see Figure 12, red spectral line) clearly show the inverted bands corresponding to the bending modes of barium sulphate at about 615 and 640 cm^−1^, as well as the combination bands located between 2500 and 1870 cm^−1^. For sample *Janini_026*, however, it is confirmed that there is indeed no barite layer, since the IR bands associated with barium sulphate are not identified (see Figure 12, blue spectral line).

**Figure 13:**
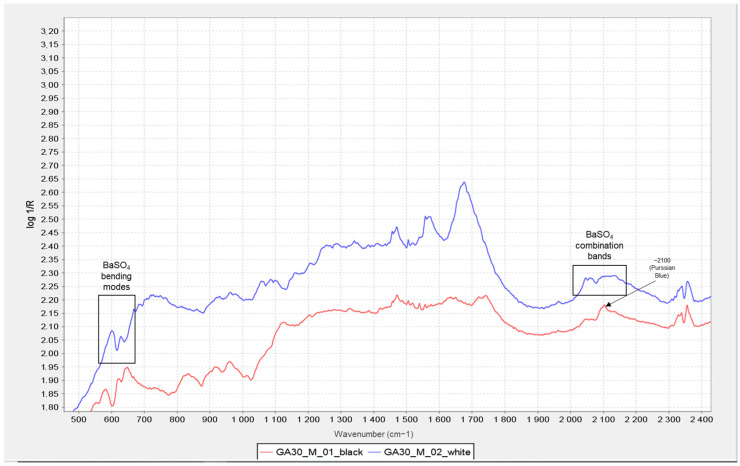
Spectra corresponding to the Dmin (blue) and Dmax (red) regions of the bromoil sample *Janini_030* with a barite layer. It can be seen how the relative intensity of the barium sulphate bands in Dmax decreases with respect to the Dmin region and the typical band of Prussian blue appears at 2100 cm^−1^. Spectra obtained with the Alpha mobile spectrometer (Bruker Corporation, Billerica, MA, USA).

In the spectra obtained with the mobile spectrometer, it is again observed how, as the image density increases, the relative intensity of the described bands corresponding to barium sulphate decreases in Dmid and Dmax (see Figure 13).

Thus, according to the results presented, it is possible to identify IR bands characteristic of barium sulphate in bromoil photographic prints by means of FTIR spectroscopy in the reflection mode, which are particularly evident in the areas of minimum density of the image, while they may be hidden or have lower relative intensities in the dense areas of the image. Specifically, by correlating the data previously obtained with the optical microscope and the digital surface microscope with the results of the mobile FTIR (applied only to the Pla Janini samples), it was possible to determine that three of the eight historical samples of Joaquim Pla Janini, namely *Janini_018*, *020*, and *026*, showed no signs of barite.

**Figure 14:**
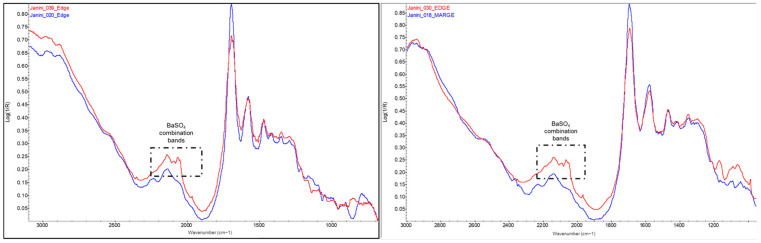
On the right, a comparison of two-point FTIR spectra corresponding to the Dmin region of a barite-coated bromoil sample (*Janini_030*, red) with one without barite (*Janini_018*, blue). On the left, comparison of two points corresponding to the Dmin area of a barite-coated bromoil sample (*Janini_039*, red) with one without barite (*Janini_020*, blue). The combination bands of barium sulphate in the range of 2500 to 1870 cm^−1^ are evident in samples *030* and *039* (with barite), whereas they are absent in samples *018* and *020* (without barite). Spectra obtained using a Nicolet™ iN10 MX desktop spectrometer (ThermoFisher Scientific, Waltham, MA, USA).

On the other hand, it was very complex or uncertain to confirm the presence of the barite layer using the Nicolet iN10 MX desktop spectrometer (ThermoFisher Scientific, Waltham, MA, USA), since the spectral range of the instrument does not allow the inverted bands at 640 and 610 cm^−1^ to be displayed, and therefore, the identification can only be made using the combination bands at 2500–1870 cm^−1^ (see Figure 14). These data were confirmed by comparison with the FTIR results from the mobile instrument.

**Figure 15:**
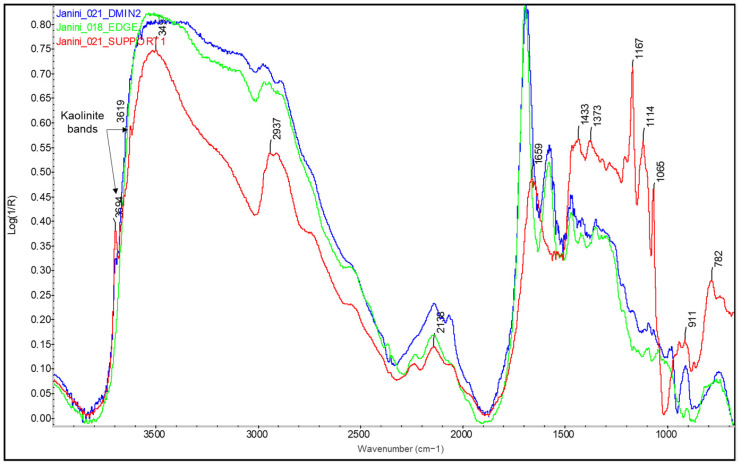
FTIR spectra of the edge region (Dmin) of the historic barite-free bromoil sample *Janini_018* (green), the Dmin region (blue), and the back of the primary support of sample *Janini_021* (red), which appears to contain a barite layer. The typical bands associated with kaolinite at 3685 and 3620 cm^−1^ from the primary support of sample *Janini_021* are observed, but there is no significant difference in the detection of cellulose from the back side between the two samples. Spectra obtained using the Nicolet™ iN10 MX desktop spectrometer (ThermoFisher Scientific, Waltham, MA, USA).

Despite the limitations of the desktop equipment for the characterisation of barite, this system has allowed the detection, in some samples, of inorganic compounds that can be attributed to the paper support, such as small traces of kaolinite (3685–3620 cm^−1^). The similar response of this compound in the spectra of the back, the front in Dmin, and the areas of the margin without ink shows that it is not part of the layers found only on the front of the copy but is intrinsic to the paper (see Figure 15).

#### 2.1.4. Oil-Based Ink

Contrary to the other compounds presented, and as mentioned at the beginning of this section, this study confirms that the oil-based ink is the only layer of a bromoil print whose components show a response in FTIR that is directly proportional to the density of the image.

Using FTIR in the external reflection mode it is possible to confirm the presence of drying oils in the bromoil prints, mainly in the Dmax areas, thus confirming the oily nature of the ink with one of the two spectrometers used in the study, although the desktop equipment has allowed a more precise identification of the bands associated with the oils and their level of polymerisation.

**Figure 16:**
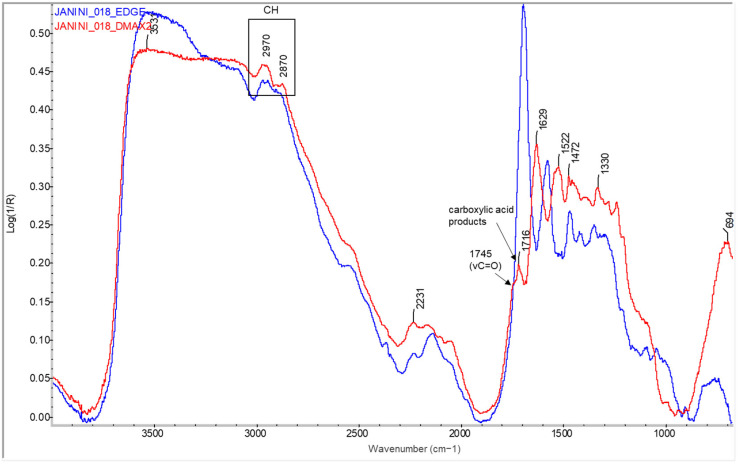
FTIR spectra of the edge area (blue) and Dmax (red) of bromoil sample *Janini_018*. Spectral changes related to the appearance of absorption bands characteristic of the drying oil present in the greasy ink can be seen at Dmax, due to the appearance of the carbonyl band at 1745 cm^−1^ and the formation of carboxylic acid products at 1716 cm^−1^, related to the polymerised state of the oil. Changes are also observed in the CH region of the oil at 2970 and 2870 cm^−1^. Spectra obtained using the Nicolet™ iN10 MX desktop spectrometer (ThermoFisher Scientific, Waltham, MA, USA).

The carbonyl band (1630–1750 cm^−1^), which is sharply defined at 1750–1740 cm^−1^ by the ester group (see Figure 16), is the most characteristic absorption band of oils [37]. This band is a clear characteristic of oil because it is the only natural organic material of the five main categories (proteins, oils, vegetable and insect resins and waxes) that has an intense carbonyl band in this region. The problem is that when mixed with certain pigments, the carbonyl band can be shifted to lower wavenumbers [32]. Other characteristic bands of oils are the aliphatic C-H bands that appear at about 1464, 1378, and 723 cm^−1^ and the C-O bands at 1240, 1165, and 1100 cm^−1^ [32,37], but in the case of compositionally complex samples, such as bromoil prints, these bands are overlapped by other compounds present in the prints, such as the protein binder, cellulose or barium sulphate, etc., making it difficult to confirm the presence of drying oils in this region (see Figure 16).

In the typical oil spectrum, the CH_2_ stretching bands are also prominent in the upper wavenumber region of the spectrum. The olefinic C-H stretching band at 3010 cm^−1^ is attributed exclusively to non-conjugated and symmetrically disubstituted cis double bonds [35], while the methylene bands are located at 2926 and 2855 cm^−1^ [32]. The visualisation of these bands is also often compromised by the superposition of bands from different compounds or spectral distortions, as will be shown below. For this reason, the detection of drying oil in bromoil samples has been mainly based on the carbonyl bands appearing in the 1630–1750 cm^−1^ region.

On the other hand, it is important to note that in order to associate the presence of esters with the image-forming material, and thus to attribute them to a print with greasy inks, these esters must be detected with a relative intensity that is directly proportional to the density of the image, since the amount of ink, and therefore the concentration of drying oil, is also proportional. Otherwise, the presence of esters in a photographic print could be circumstantial (e.g., possible retouching) and not due to the specific photographic process. In other words, the intensity of the bands corresponding to the oils is greater in the areas of greater image density (Dmax) and may be imperceptible in the lighter areas of the image (Dmin), even if they contain a certain amount of ink. This is because, in these Dmin areas, the compounds of the lower layers appear between the ink-free areas, so that they are detected clearly and with greater intensity than in the areas where they are covered by the ink layer (Dmax). It is in these Dmid and Dmax areas that the typical oil bands begin to be distinguished.

For example, Figure 16 shows the Dmin and Dmax spectra of a Dmin region of the *Janini_018* sample. The Dmin or edge spectrum (blue spectral line) consists of the typical bands of the protein binder and some coming from the lower cellulose. Comparing the Dmax spectrum (red spectral line), a new rather broad band appears centred at 1716 cm^−1^, consisting of a strong shoulder at 1745 cm^−1^ related to the carbonyl band characteristic of oils. The peak protruding from the centre of the band at 1716 cm^−1^, according to the studies carried out by Meilunas et al. [35], suggests the formation of carboxylic acid products, components associated with oxypolymerised oily materials. Meilunas et al. [35] also point out that the absorption peak centred at 1776 cm^−1^ is useful for identifying aged oil samples, since other materials, including pigments, are generally transparent in this region of the spectrum. Other authors, such as Lazzary and Chiantore [38] and Derrick et al. [32], who have studied the structural and spectral changes in linseed oil during drying and aging, also point to a broadening of the carbonyl absorption (1850–1650 cm^−1^) and the formation of additional ketones, esters, and acid carbonyls (1750–1700 cm^−1^). In the present sample, the back at 1776 cm^−1^ of the polymerised oils mentioned in the references is not very prominent (see Figure 16), although it may be hidden under the broad carbonyl band, which appears as a prominent back at 1745 cm^−1^. It should be noted that in the spectra of the reference literature [35], this back at 1770–1780 cm^−1^ is also not pronounced but rather weak, similar to the experimental spectrum of sample *Janini_018* (see Figure 16).

This pattern is clearly repeated in all the bromoil prints examined, although the clarity of the oil absorption bands obviously varies according to the point, area, and sample analysed.

As mentioned above, it is difficult to distinguish between the aliphatic C-H and C-O bands in the 1460–700 cm^−1^ region due to the bands of other compounds present in the sample, such as gelatine and cellulose. Although these materials are found in lower layers and their absorptions are clearly influenced by the presence of a thick layer of oily ink, their bands eventually predominate and are completely mixed with those of the oil in this region of the spectrum. It should also be noted that other materials may be involved, such as the pigment itself, additives, fillers, etc. Finally, in the region between 3000 and 2850 cm^−1^, it is difficult to distinguish significant absorptions that may be associated with the drying oil, such as the prominent hydroxyl/hydroperoxide band between 3600 and 3400 cm^−1^ [35,38], mainly due to the confluence of bands from several compounds. However, a smooth curve is observed in this region, as well as a slight spectral shift at 2969 and 2870 cm^−1^ between the spectrum of the edge region (see Figure 16, blue spectral line) and the Dmax region (see Figure 16, red spectral line), bands that may be associated with the C-H stretching modes of the oil.

**Figure 17:**
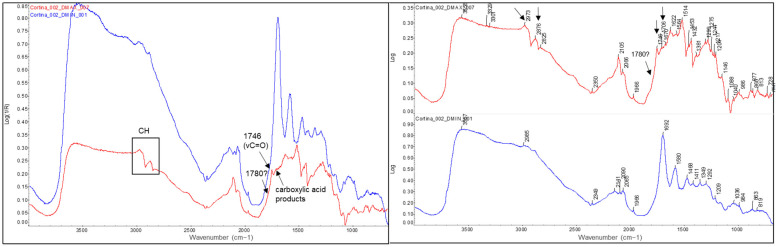
FTIR spectra of the Dmin (blue) and Dmax (red) regions of the bromoil sample *Cortina_002*. The carbonyl absorption bands at 1746 cm^−1^ and the CH bands of the oil at 2972 and 2876 cm^−1^ can be seen. Spectra obtained using a Nicolet™ iN10 MX desktop spectrometer (ThermoFisher Scientific, Waltham, MA, USA).

A very similar analytical profile was obtained in the samples to the bromoil of Josep Cortina Molist. In the figure below (see Figure 17), the spectrum of the Dmin region (blue spectral line) and Dmax region (red spectral line) corresponding to sample *Cortina_002* are shown. In the spectrum of the Dmax region, the carbonyl band of the oil appears centred at 1746 cm^−1^ and a slight shoulder seems to start to form at ~1780 cm^−1^. In this case the band at 1715 cm^−1^ is not clearly distinguishable, probably because the bands in this region appear very close together and practically merge with the absorption bands of the gelatine, although a small peak can be seen that stands out near 1715 cm^−1^. The fact that the carbonyl band appears perfectly centred at 1746 cm^−1^ and the shoulder at 1715 cm^−1^ of the carboxylic acids is not so present indicates that there is perhaps a lower level of polymerisation of the oil compared to the *Janini_018* sample seen previously. Again, the appearance or definition of bands at 2972 and 2876 cm^−1^ can be seen, which can be related to the CH vibrational modes of the oil. However, in this sample, the appearance of oxidation products with hydroxyl characteristics (3600–3000 cm^−1^) seems to be better distinguished.

**Figure 18:**
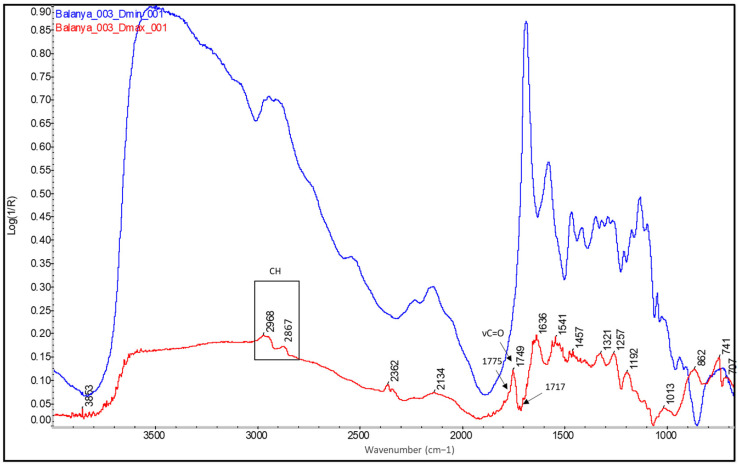
Dmin (blue) and Dmax (red) area spectra of the contemporary bromoil sample *Balanya_003*. The Dmax area spectrum clearly shows the carbonyl band of the oil at 1748 cm^−1^, accompanied by a small shoulder at 1775 cm^−1^ and a small peak at 1717 cm^−1^, absorptions associated with oxypolymerised lipid materials. C-H stretching bands are also detected at 2968 and 2867 cm^−1^. Spectra acquired on a Nicolet™ iN10 MX desktop spectrometer (ThermoFisher Scientific, Waltham, MA, USA).

Finally, it is interesting to compare the spectra of the original historical samples from the mid-20th century with those of the more recent bromoil samples by Jaume Balanyà. Despite the fact that the latter are almost 14 years old, the spectral differences due to the polymerisation, oxidation, and aging reactions are obvious when compared with a sample that is almost 90 years old, such as the *Janini_018* print. If we look at the Dmax spectrum of the *Balanya_003* sample (see Figure 18), we can see some small changes, especially in the region between 1790 and 1710 cm^−1^. In this case, the typical carbonyl band of drying oils appears more angular and perfectly defined, centred at 1749 cm^−1^. Next, associated with the formation of carboxylic acid products, is a very small peak at 1717 cm^−1^. However, this is almost imperceptible, especially when compared to the sharp peak of the *Janini_018* Dmax spectrum (see Figure 16) or the broader peak at 1715 cm^−1^ of the *Cortina_002* sample (see Figure 17, red spectral line), which is consistent with a lower oxidative polymerisation of the oil compared to the historical samples. There is also a small peak at 1775 cm^−1^. In addition, the presence of oxidation products with hydroxyl characteristics (3600–3000 cm^−1^) is not identified, unlike the older samples where, although they are mixed with bands of other compounds due to the position of the absorptions, it is possible to perfectly guess the curvature of the spectral line between 3600 and 3400 cm^−1^, which on the other hand does not appear in the Dmax spectra of the contemporary samples (see Figure 18, red spectral line).

#### 2.1.5. Pigments

Finally, reflection-mode FTIR spectroscopy has proved useful in detecting certain pigments present in the sample images, particularly using Bruker Corporation’s mobile Alpha instrument (Billerica, MA, USA), thus allowing the print to be characterised as a pigmented photographic print.

The main pigments detected were Prussian blue or ferric ferrocyanide (Fe_4_[Fe(CN)_6_]_3_) and earth pigments composed of iron oxides, detectable thanks to the Fe-O and Fe-OH vibrations that make them identifiable with FTIR.

**Figure 19:**
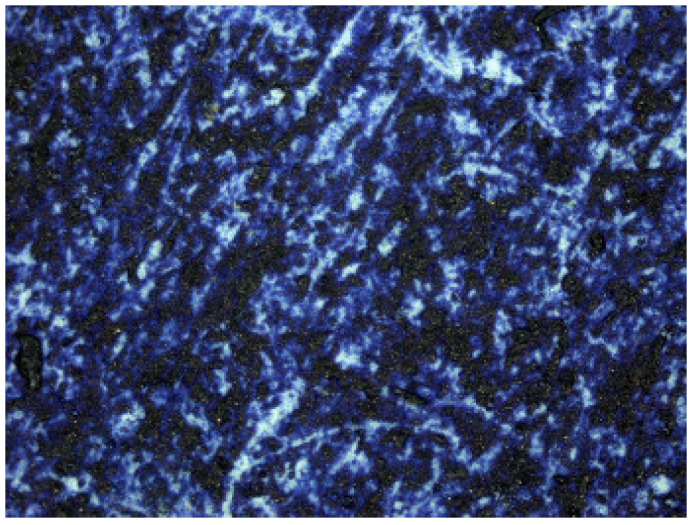
Cortina_003_Dmax 1 (6) 180× (digital surface microscope).

**Figure 20:**
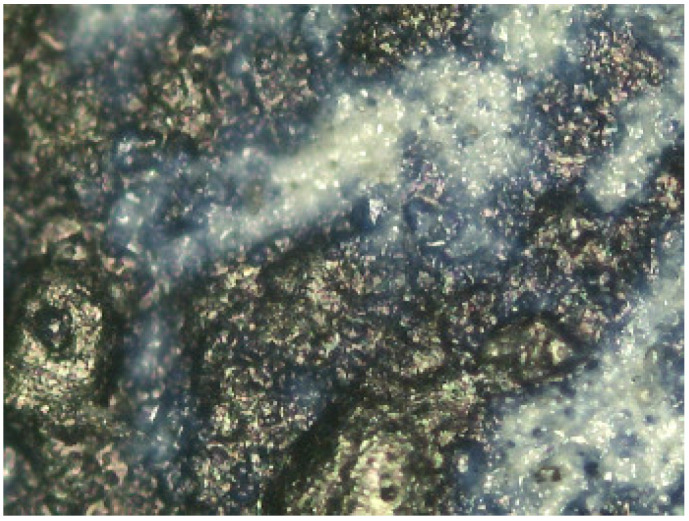
Cortina_003_Dmax 100× (optical microscope).

Prussian blue has been detected in almost all the samples, except for the three contemporary bromoil samples by Jaume Balanyà, which show a warm black image, and the historical samples *Janini_018* and *Janini_039*, both of which were produced with a clearly orange-brown pigment. In the great majority of samples where the Prussian blue pigment has been detected, it is present as a secondary pigment in the black ink, i.e., as an additive to give more intensity and contrast with the warm tone of the black pigment. In other samples with more bluish or greenish tones, such as *Cortina_003* and *Janini_028*, it has been visualised microscopically as a single colour within the palette that forms the images (see Figure 19 and Figure 20).

**Figure 21:**
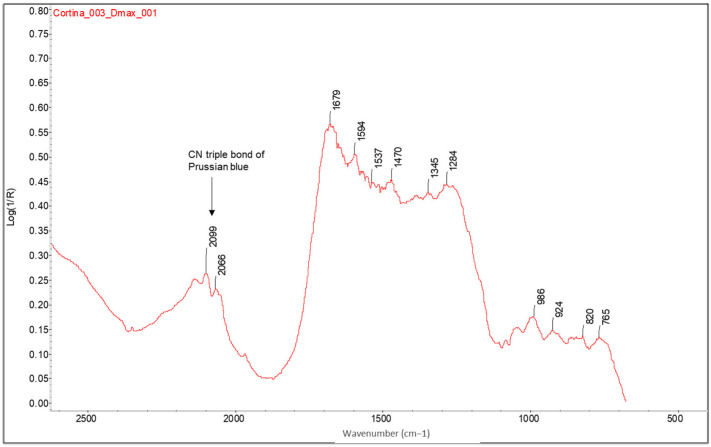
Spectrum of the Dmax region of the bromoil sample *Cortina_003*, where the characteristic band of the CN triple bond of Prussian blue is identified, in agreement with the blue-tinted image of the copy as observed in the microscope images. Spectrum obtained using a Nicolet™ iN10 MX desktop spectrometer (ThermoFisher Scientific, Waltham, MA, USA).

Using FTIR spectroscopy, Prussian blue is an easily identifiable pigment because it shows a strong absorption band at around 2100 cm^−1^, due to the triple bond CN (see Figure 21). It also usually shows a band at 500 cm^−1^ and two weak bands at ~608 and ~1420 cm^−1^ [39], but these are difficult to distinguish in the recorded spectra due to the superposition of bands from other compounds in these regions of the spectrum.

**Figure 22:**
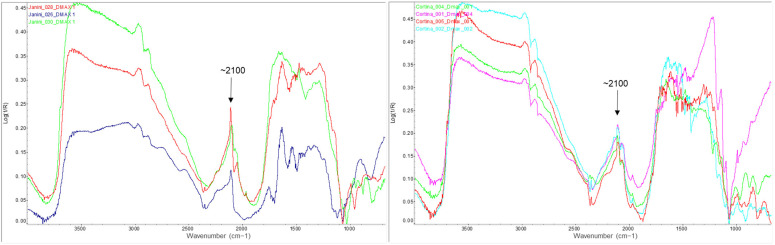
FTIR spectra of the Dmax regions of the bromoil samples *Janini_026* (blue), *028* (red), and *030* (green) on the left and *Cortina_001* (pink), *002* (blue), *004* (green), and *005* (red) on the right, where the strong absorption band at ~2100 cm^−1^ of ferric ferrocyanide (Prussian blue) is clearly visible. Spectra obtained using a Nicolet™ iN10 MX desktop spectrometer (ThermoFisher Scientific, Waltham, MA, USA).

Further FTIR spectra are shown below, taken from the areas of maximum density in the image of samples where ferric ferrocyanide has also been detected thanks to the clearly visible absorption peak at ~2100 cm^−1^ (see Figure 22). In addition, sample *Janini_028* contains a higher amount of this pigment, which can be deduced from the more intense signals it shows (see Figure 22, left, red spectral line).

Iron oxide pigments have been found mainly in association with those bromoil images that have a distinctly umber tone, although the chromatic range varies from more yellowish light brown tones to darker or reddish tones. This type of pigment was found in samples *Janini_018*, *020*, *021*, *039*, and *051*. The problem with umber or brown pigments is that, although FTIR spectroscopy has been used to verify the presence of one or more iron-oxide-based pigments in these images, it has not been possible to identify the specific pigments of which they are composed by FTIR spectroscopy, which would require a combination of other analytical techniques, such as Raman spectroscopy or X-ray fluorescence. This study has therefore been able to demonstrate the potential but also the limitations of FTIR in identifying pigments.

**Figure 23:**
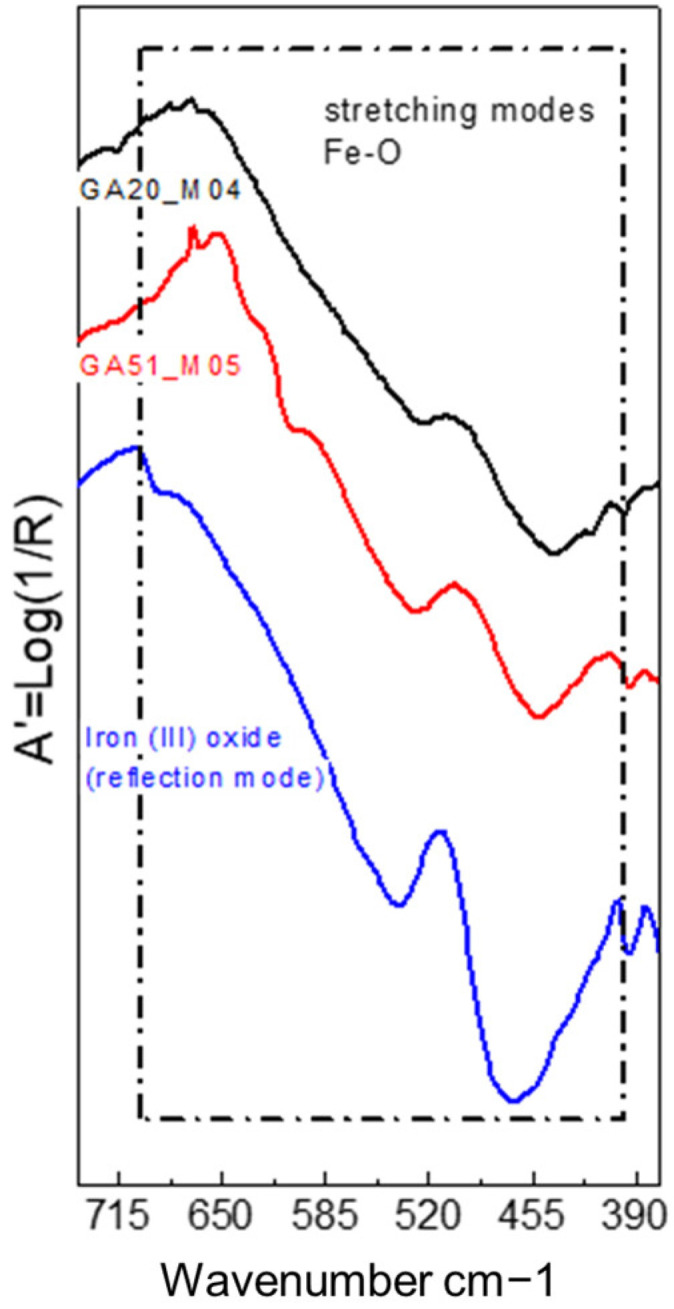
Using reflection mode FTIR spectroscopy, iron (III) oxides are identified from two inverted bands in the 670–400 cm^−1^ range corresponding to the Fe-O stretching modes (see comparison of reflection mode spectra recorded for the corresponding reference compound (blue line) and for two of the samples analysed (black and red). Spectra obtained with the Alpha mobile spectrometer (Bruker Corporation, Billerica, MA, USA).

**Figure 24:**
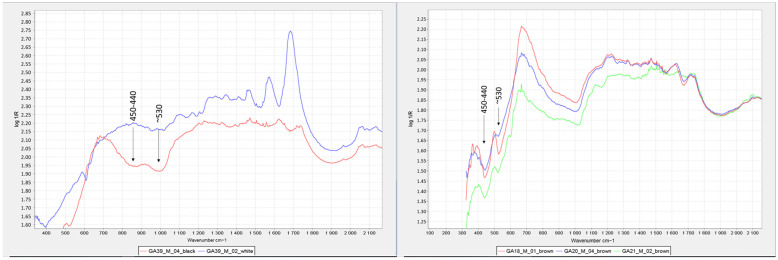
Location of the inverted bands of the stretching modes (Fe-O) of the ferric oxides corresponding to the umber pigments detected in the bromoil images of the *Janini_039*, *018*, *020*, and *021* samples. The FTIR spectrum on the left shows a comparison between a Dmin (in blue) and Dmax (in red) area; note that the bands located at 530 and 450–440 cm^−1^ appear only in the Dmax area. Spectra obtained with the Alpha mobile spectrometer (Bruker Corporation, Billerica, MA, USA).

FTIR spectroscopy allows the detection of iron oxide pigments from two absorption bands located at the edge of the spectral range corresponding to low wavenumbers (~530 and ~440–430 cm^−1^) (see Figure 23 and Figure 24). However, these bands are not characteristic enough to identify the pigment in question [40], and in addition, the infrared bands of ferric oxides and hydroxides are outside the accessible range of some instruments [24], such as the Nicolet iN10 MX desktop spectrometer (ThermoFisher Scientific, Waltham, MA, USA). Therefore, iron oxides were only identified in the samples analysed with the mobile instrument Alpha (Bruker Corporation, Billerica, MA, USA). On the other hand, when using the reflection mode, these bands are distorted by specular (surface) reflection, producing inverted bands or residual peaks located between 600 and 400 cm^−1^ [40] (see Figure 23). In the case of the bromoil samples analysed, these bands were more specifically located at wave numbers 530 and 450–440 cm^−1^ (see Figure 23 and Figure 24).

**Figure 25:**
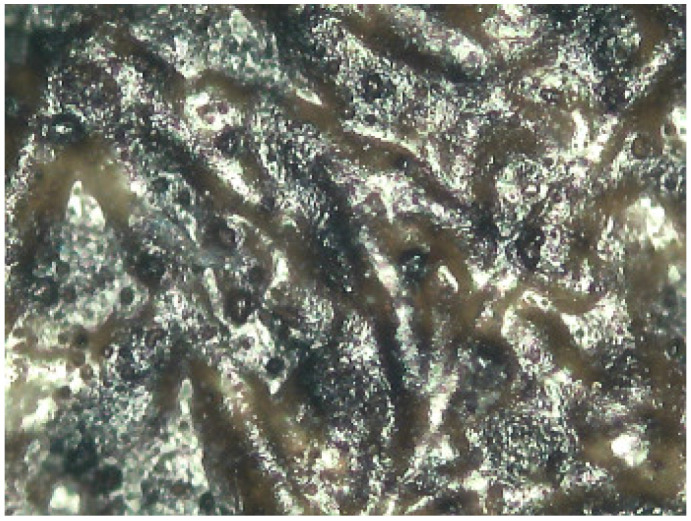
Janini_020_Dmax_100× (optical microscope).

**Figure 26:**
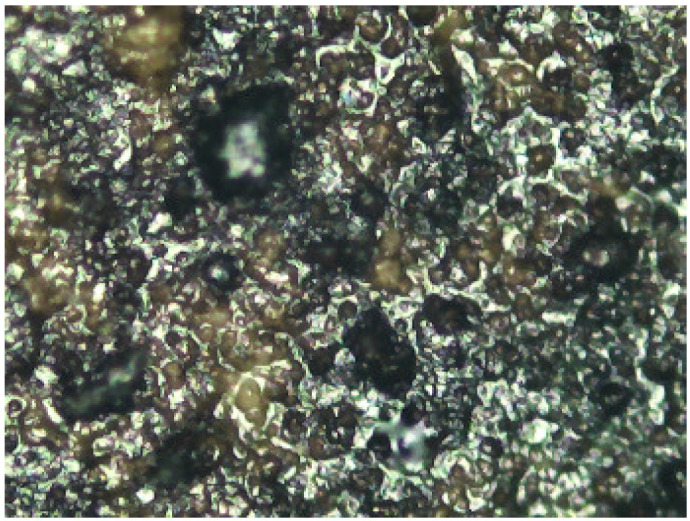
Janini_051_Dmax_100× (optical microscope).

In the case of both iron oxide pigments and Prussian blue, the characteristic IR absorption bands appear parallel to the increase in image density, i.e., in relation to the areas of maximum image density. This confirms that these are compounds involved in image formation. The presence of pigment particles with these characteristics was also previously confirmed by microscopy (see Figure 25 and Figure 26).

### 2.2. Discussion

In the current study, we have succeeded for the first time in characterising the main components of all the layers of bromoil prints via FTIR spectroscopy. Given the inherent heritage value of these works, the methodology developed is non-destructive and non-invasive.

The paper proposes a study methodology through which FTIR spectroscopy in external reflection mode emerges as a highly valuable technique for identifying bromoils prints, characterising them, gaining a deep understanding of their composition and structure, and even detecting mechanisms of alteration and/or aging of their components.

As a result of applying this methodology, the possibilities and limits of the application of FTIR in the study of bromoils prints are unveiled, considering the capabilities of two types of spectrometers, one desktop and one mobile. The results of the study display the compounds that can be detected, as well as the conditions under which these, when detected in a photographic print, can be considered indicators or evidence for the identification of bromoils prints.

The basis of the proposed methodology lies in the identification system introduced by James Reilly in 1986 [41], which classifies historic photographic prints, specifically from the 19th century, into copies of one, two, or three stratigraphic layers depending on how the fibres of the paper support are observed from the front through different image areas. With the purpose of employing some of Reilly’s methods for the study and identification of photographic copies, we have adapted to the bromoil prints analysis methodology two fundamental issues: on one hand, discerning measurement areas based on image density and, on the other, interpreting the results of the spectra based on the stratigraphic structure of the sample. Adopting these criteria allows us to demonstrate the potential to recognise the qualities of the different compounds present in the copy and to relate them to their stratigraphic position, thus establishing a deeper interpretation of the composition and structure of the bromoil prints.

The combination of these two pieces of information—composition and structure of the layers—is indispensable not only for understanding the original but also for its identification as a bromoil print.

Thus, the method used has proven to be very effective in obtaining optimal results, so it can be said that it significantly contributes to the methodological issues for future studies of bromoil copies—and quite likely of fatty positives in general—using spectroscopic techniques.

The results have demonstrated that all those materials inherent to the image, that is, the oils and the pigments that make up the oil-based ink, have been detected in intensity proportional to the image density, mainly in the Dmid and Dmax areas. In contrast, all those compounds associated with the original photographic support, such as the protein binder, the barite layer, and the cellulosic substrate, which are located in layers beneath the greasy ink layer, have been detected with inverse proportion to the image density. This is due to the greasy ink acting literally as a physical layer that, as it increases in concentration in the areas of medium and maximum image density, covers and hides the FTIR signals coming from the lower substrates. This aspect is characteristic of oil-based ink copies and should, therefore, be considered as a key to the identification of these works. This is crucial to avoid incorrect identifications that may result from erroneously attributing the spot detection of oily materials on the surface of a photograph made by another procedure—such as possible retouches where paints containing drying oils are used—to the presence of a greasy ink layer that forms the image, characteristic of a bromoil copy.

Thus, when analysing a bromoil copy, measurements made in areas of maximum image density (Dmax) mainly allow detecting and recognising the materials that form the image, such as pigments and drying oils that make up the oil-based ink, while measurements in areas of minimum image density (Dmin) detect more intensely and clearly the characteristic bands of the materials coming from underlying layers (barite layer, photographic binder, and paper support), as these areas of the image are not hidden under the greasy ink layer, as happens in Dmax. In the case of cellulose, it remains almost hidden even in the Dmin areas due to the overlap of the characteristic bands of the protein binder. If possible, it is also advisable to perform measurements at points of medium image density (Dmid) to study more deeply the changes generated between image areas. Finally, if the sample allows, it is recommended to conduct tests on the back of the primary support to rule out materials specific to the paper support that may interfere in the detection of materials of interest when analysing the sample from the front.

**Figure 27:**
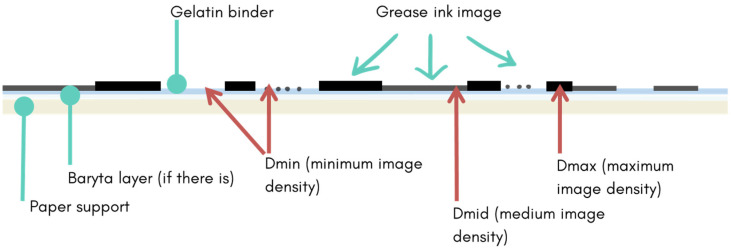
Graphic representation of the stratigraphic structure of a bromoil print. At least three layers are distinguished: the paper substrate, the barite layer (if present), the gelatine binder, and the layer of greasy ink that forms the image. The areas of Dmin correspond to the areas with the minimum ink content, the Dmid areas contain a medium density of the image, and the Dmax areas correspond to the blacks or points of maximum ink density.

This study demonstrates that FTIR spectroscopy in external reflection mode is a technique capable of characterising the major components of each of the layers that constitute bromoils and their location in the work: the cellulose of the paper, the barite layer, the gelatine layer, the drying oils, and certain pigments of the image-forming layer. Thus, the results obtained both from close examination under the microscope and FTIR spectroscopy allowed us to propose a clear representation of the stratigraphic structure of the bromoil copies (see Figure 27).

On one hand, the results of our study demonstrate that in bromoil, the image-forming material, namely the greasy ink, is not located inside the gelatine, as is the case in most non-fatty photographic processes with a protein binder. Instead, it is situated in a top layer, in line with other reference sources [8]. Therefore, the gelatine cannot be considered a colloid that suspends the particles forming the image, whether silver or pigments, as in other non-fatty and silver pigmentary photographic processes, but rather a layer that functions similarly to a matrix at the time of image creation, when the oil-based ink is manually incorporated into the copy, adhering to it based on the degree of wetness of the gelatine, inversely proportional to the degree of hardening suffered during the prior bleaching process, according to the silver concentration of the original bromide [7,42,43,44]. From these results, we propose to reconsider the use of the terms *colloid* or *emulsion* when talking about the gelatine layer in bromoils (terms widely used for pigmentary and silver copies). Considering the function this protein layer has in bromoils, it is suggested to better understand it as a *matrix* or *intermediary layer.*

This conceptual and terminological issue holds pivotal relevance in the characterisation of bromoils through instrumental analysis as, as shown by the results of this study, the location of the ink layer as an independent stratum that overlays the rest of the layers directly affects how the other materials of the work inherent to the photographic support are detected, namely, those situated in layers beneath the ink, constituting this phenomenon a key identification factor of bromoil copies.

On the other hand, this study reveals the existence of bromoil prints without a barite layer, which cannot be considered exceptional cases. Of the eight historic samples from Joaquim Pla Janini, analysed with the Alpha mobile spectrometer (Bruker Corporation, Billerica, MA, USA), three prints without a barite layer were detected, coinciding with the results of the microscopic study. This indicates that the presence of barite is not an indicator for the identification of the bromoil technique. Moreover, the microscopic examination also did not provide evidence of a barite layer in the three contemporary bromoil prints, information that was confirmed by the testimony of the author himself, who provided samples and the exact brand of the photographic paper used, Kentmere^®^ Document Art (Balanyà, personal communication, June 2019). These observations help clarify the current contradictions that appear in the literature on this topic where, on one hand, bromoil copies are included within the category of two-layer photographic processes, alongside carbon and gum dichromate prints [9], overlooking the fact that many bromoil prints contain a barite layer. However, in other references [8], the presence of this barite layer is considered one of the keys to the identification of bromoil prints. The use of FTIR spectroscopy for the analysis of heritage collections of bromoil prints has served to deepen the recognition of the compounds forming their structure, especially in terms of detecting the barite layer, which may be often absent. This allows us to suggest that the presence or absence of the barite layer can no longer be considered an identification key for bromoil copies. This study contributes to the generation of new knowledge of bromoil prints verified through the study of a heterogeneous collection of samples by means of FTIR analysis.

Therefore, it is important to consider that the characterisation of bromoil prints using the visual recognition system by layers must consider the possibility that these works have four layers in cases where they include the barite layer and three layers when using a paper without this intermediate stratum. The classification of bromoil positives as three- or four-layer photographic prints should also consider the greasy ink layer as an independent stratum.

It should be mentioned, therefore, that during instrumental analysis, it is important to precisely select the measurement points of the copy to maintain rigor in the interpretation of the spectra based on the presence of the ink layer in each analysed area (Dmin, Dmid, and Dmax). During this study, it has been verified that conducting the analyses with a spectrometer equipped with an attached microscope significantly increases the accuracy and reliability of the results, especially in the case of complex photographic samples like those analysed, where the distribution of materials can vary significantly across the surface.

In this article, it has been demonstrated that the external reflection mode is highly suitable for characterising bromoil prints using FTIR spectroscopy because, on one hand, from a methodological perspective, it is less invasive than the ATR mode as it avoids contact with the instrumentation, and on the other hand, this mode has proven useful for achieving the identification of the compounds in these works. The main drawback of using FTIR spectroscopy in external reflection mode is related to the interpretation of spectral data, which can exhibit significant distortions in bands shape, absorption frequency, and intensity compared to transmittance spectra.

These anomalies, produced by various factors such as absorption indices (*k*) and refraction indices (n) and surface roughness, which affect the extent of contributions from specular (or surface) and diffuse (or volume) reflection [28], can lead to Reststrahlen bands (or inverted bands) when the sample contains a compound with *k ≫ 1* [28,36]. This last distortion occurs frequently in bromoil samples in the analysis of the barium sulphate present in the barite layer and in iron oxide pigments.

The type of equipment used can also contribute significantly to the spectral response. Generally, the optical geometry of portable or mobile infrared fibre optic systems tends to maximise specular reflected light by working with an angle of incidence equal to the reflected angle [24]. However, in the infrared reflection measurements of works such as the photographic prints analysed, both surface reflection and volume reflection are generally active, affecting the spectral characteristics depending not only on the optical properties of the materials but also on the surface roughness. Regarding morphological properties, surfaces with particle sizes larger than the wavelength of infrared radiation provide a greater amount of specular light, while rougher surfaces, with particle dimensions similar to the wavelength of infrared radiation, mainly generate diffuse reflected radiation [24]. Optical properties, in terms of absorption and refraction indices, will influence the degree of light penetration and, therefore, the contribution of surface and volume reflection.

Consequently, in the analysis of complex samples, such as bromoil prints, where surface properties are very heterogeneous, different types of distortions may coexist in the same spectrum due to the derived shape, Reststrahlen effect, and intensity enhancement, making it impossible to apply Kramers–Kronig and Kubelka–Munk corrections [45], significantly complicating their interpretation.

In this study, this was particularly recurrent in the identification of barium sulphate from the barite and iron-oxide-based pigments. In the bromoils analysed, the cellulose and gelatine bands of the spectra obtained by reflection were comparable without much difficulty to the known bands of these materials in ATR mode.

Finally, it is particularly relevant to note the advantages and limitations that have been detected during the use of different types of instrumentation, as they have directly influenced the results. In the case of desktop spectrometers, first, the geometry or design characteristics of the equipment must be considered, not only to properly position the work in the spectrometer but also for the selection of the areas and measurement points of the samples. These devices are designed for analysing small samples, which can present difficulties for the light source to access more internal areas of the work, clearly restricting the selection of analysis points. Partly, this was one of the reasons why access to a portable device was sought to overcome this limitation inherent to the desktop equipment.

Moreover, and very importantly, the components (different sensitivities in varied spectral ranges) and the configuration of the spectrometer that may influence the extent of the spectral range must be considered. That is, depending on the limit set for the spectral range, IR bands of certain compounds that may be present may not be visible because their absorptions fall outside the established range. This is especially relevant when using the external reflection mode for certain compounds, such as with barium sulphate or iron oxides, because spectral distortions and band shifts occur in “unusual” ranges of the spectrum, which can lead to erroneous conclusions. In the present study, this circumstance occurred with the desktop equipment, with which the inverted bands associated with the mentioned compounds, appearing in wavenumber ranges of the lower range, could not be observed because the limit of the spectral range of this equipment was established around 700 cm^−1^. The limit of the spectral range, however, is not necessarily directly related to the model (desktop or mobile) but rather to its features and configuration.

To conclude, although all major compounds have been detected with both devices, the desktop equipment has shown superior resolution, allowing greater precision in identifying minor bands associated with secondary compounds or physicochemical phenomena related to the state of conservation and aging of the samples, such as the appearance of absorptions that can be related to the degree of the oxy-polymerisation of the drying oils in the greasy ink.

## 3. Methodological Part

### 3.1. Photographic Artifacts

This study is based on 16 photographic bromoil samples, of which 13 are original historic prints can be dated between the late 1920s and 1960s, and 3 are contemporary prints produced in 2009–2010 (see Table 1 and Table 2). The selection of works studied constitutes a sufficiently varied sample from both technical and material perspectives, regardless of the production era of the copy, which inherently introduces an implicit technical and material variable. The study includes bromoil prints composed of different tonalities and types of inks, some combined in the same work, and a significant variety of photographic supports of different formats, finishes, and surface textures.

The historic bromoil samples come from two different collections and correspond to two authors of Spanish nationality. These copies were produced during the period of greatest expansion of the bromoil technique in Spain, covering the different phases that characterise the pictorialist movement in this country [1,3,46,47,48]. On one hand, the sample includes eight bromoil prints produced by the bromoil master Dr. Joaquim Pla Janini (1879–1970), considered the foremost reference for the bromoil technique in Spain and internationally recognised [3,47]. On the other hand, the historic bromoil sample also includes a total of five works by Josep Cortina Molist (1918–1995), an amateur photographer who was associated with the Agrupació Fotogràfica de Catalunya (A.F.C.), of which Dr. Pla Janini was the director during the years from 1927 to 1930, a period of peak activity for the A.F.C. [49]. In both cases, the selected works come from the private collection of the heirs of the aforementioned photographers.

The historic bromoil prints are an authentic reference: analyses from original works allow for a reliable understanding of the techniques and materials used during the height of pictorialism, as well as their evolution throughout the 20th century. This not only contributes to a more robust and reliable research methodology but also provides data on how these prints may have changed or deteriorated over time.

**Figure 28:**
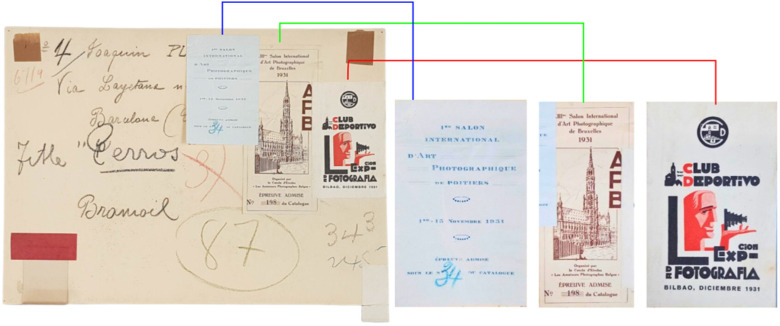
Participation labels from salons and exhibitions located on the back of the bromoil work *Janini_030*, including the I Salon International d’Art Photographique de Poitiers (November 1931), the XIII Salon International d’Art Photographique de Bruxeles (1931), and the exhibition organised by the Club Deportivo de Bilbao (December 1931).

All the selected works of Dr. Joaquim Pla Janini have been corroborated as bromoil prints by the information written on the backs of the works, primarily annotations made by the artist himself, as well as stamps and labels from exhibitions and salons where the photographer presented the works (see Figure 28). The selected works from the collection of Josep Cortina Molist are recognised as bromoils because of the direct testimony of the artist’s son, the person in charge of the collection, who has loaned the works for the study.

The technical excellence of Joaquim Pla Janini’s works is internationally recognised as a benchmark of high artistic quality in the bromoil process. This is supported by many of his contributions to specialised magazines of the time such as *Sombras*, *Luz*, *La Fotografía*, *El Progreso Fotográfico*, *Revista Fotográfica*, and *Arte Fotográfico*, the monograph dedicated to his person in the magazine *Art de la Llum* from October 1934 [50], or his participation in the first exhibition at the Parés Gallery in Barcelona in April 1928 or the I International Salon organised by the Agrupació Fotogràfica de Catalunya in 1929 [51], demonstrating his reputation and recognition as a leading figure of the pictorialist movement. His continued practice of bromoil reflects an artistic trajectory that spans various evolutionary stages of pictorialism and is representative of the materials and general procedures used in bromoil production in other European countries, as evidenced by the documentation of his private collection and the consultative sources preserved there, including English, German, and Italian manuals [7,52,53]. In fact, it was common for Catalan and Spanish bromoilists to draw information from manuals from other countries, especially England, Germany, and Italy. Moreover, there are very few examples of manuals and guides in Spanish and even fewer in Catalan. To give another example, among some of the manuals preserved by Josep Maria Casals i Ariet, one of the leading exponents of bromoil transfer and pictorialism in Catalonia, are Dr. Mayer’s manual [7] and the publication by Symes [53]. In addition, one of the most important magazines of the time in the photographic field, *El Progreso Fotográfico*, was the translated edition of the famous magazine directed by Professor Rodolfo Namias, *Il Progresso Fotografico*, published in Turin, Italy.

On the other hand, it is very interesting to include part of the production of an amateur photographer like Josep Cortina Molist, who serves as the counterpart to Joaquim Pla Janini and represents the entire collective of enthusiasts for artistic photography that so characterised the associativism of the era, closely linked to the pictorialist movement. Like so many other amateur photographers of the time, Cortina also participated in the activities and salons of photography so common in that era, and his bromoil photographs connect him with the pictorialist aesthetic. Like many other enthusiasts, Mr. Cortina Molist did not achieve the recognition within the artistic circles that he probably deserved, while his works demonstrate a great mastery of photographic technique. In fact, the associativism of the time allowed amateur authors to access the knowledge of bromoil process that was disseminated by the most important authors in Europe, and therefore, it is plausible to think that they frequently used materials and methods similar to those used by the Spanish and European masters of the bromoil process.

The three contemporary bromoil prints selected for this study were created by the renowned photographer Jaume Balanyà, born in Barcelona in 1949. He is a professional photographer with 50 years of experience who has participated in exhibitions, won awards, carried out reportages, and published several books [54,55,56] specialising in old processes, such as salt paper, cyanotype, and bromoil. The bromoil prints loaned for this study are part of a series of 31 bromoil photographs that were exhibited for the first time in 2010 at the Altair bookstore and, subsequently, at the Saleta of Casa Golferichs in Barcelona during January and February 2011.

Incorporating contemporary professional-quality bromoil samples into the study provides the opportunity to understand the evolution of the methods and materials used in a photographic process that has nearly disappeared. On the other hand, they serve as a comparative reference to the historic copies to study the changes derived from the aging processes that affect bromoil prints and how these are reflected in the results of the FTIR analyses.

### 3.2. Experimental Part

Analyses using Fourier transform infrared spectroscopy (FTIR) were carried out in external reflection mode with two different devices, a desktop spectrometer and a mobile spectrometer.

Measurements with the desktop equipment were conducted at the Scientific and Technological Centers of the University of Barcelona (CCiTUB) (Barcelona, Spain) using a ThermoFisher Scientific^®^ (Waltham, MA, USA) Nicolet iN10 MX spectrometer with an imaging microscope, equipped with a liquid nitrogen cooled mercury-cadmium-telluride (MCT) detector, KBr beam splitter, and globar light source. Measurements were carried out in external reflection mode, with an aperture of 100 μm × 100 μm, in the range of 4000 to 675 cm^−1^, with a resolution of 4 cm^−1^ and 64 scans, and spectra were plotted as log(1/R).

Analyses conducted with the mobile equipment were performed with instrumentation provided by the MOLAB program and were made with an Alpha mobile spectrometer (Bruker Corporation, Billerica, MA, USA), which contains a high-sensitivity pyroelectric DLaTGS (deuterated L-alanine-doped triglycine sulphate) detector at ambient temperature. External reflection of an area approximately 5 mm in diameter was measured in a spectral range of 7500 to 375 cm^−1^ and a spectral resolution of 4 cm^−1^ over 300 scans, and spectra were plotted as log(1/R).

In the resulting spectra from the analyses of the bromoil prints, the Kramers–Kronig (KK) correction was not viable due to the combined response of surface and/or volume reflection attributable to the material, morphological, and surface complexity of the samples [24]. The heterogeneous nature of the bromoil prints, which includes variations in texture, material composition, and overlapping layers, significantly complicates the application of the Kramers–Kronig relations, a correction that requires a more homogeneous, shiny/polished (highly specular sample response) medium, which limited the use of the KK algorithm in this case [14,25].

For processing FTIR spectra obtained with the Nicolet iN10 MX equipment, Omnic 9.2.86 software was used, and for the spectra collected with the Bruker^®^ Alpha mobile equipment, MOVIDA (MObile laboratory VIsualization Data) software was used [57].

Seven of the eight works by Janini were analysed using both spectrometers (desktop and mobile), except for *Janini_051*, which was analysed only with the mobile equipment. The works by Cortina and Balanyà were studied only with the desktop spectrometer. The locations of the points analysed with each device were recorded on measurement maps for each work.

**Figure 29:**
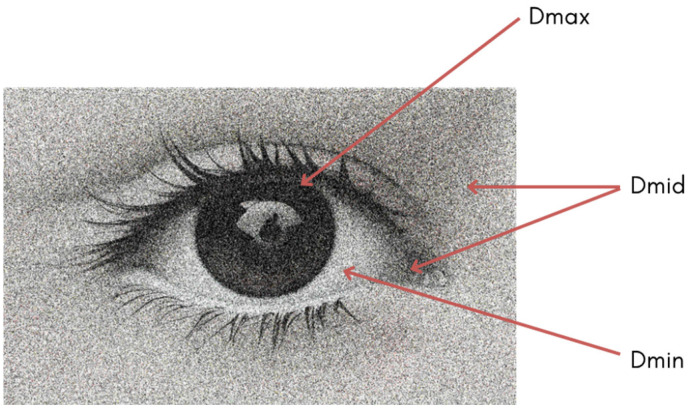
Graphic representation of the image areas of a bromoil copy corresponding to Dmax (maximum image density), which includes blacks and deep shadows with the maximum concentration of pigment from the grease ink: Dmid (medium image density), or midtones/grays, which have varying amounts of pigment (grease ink) proportional to the image density; and Dmin (minimum image density), or white areas, with little or no pigment presence. Top view.

In all the works, measurements were taken in areas of maximum image density (Dmax), equivalent to the blacks of the image, areas of minimum image density (Dmin), corresponding to the whites or highlights, and areas of medium image density or mid-greys (Dmid) (see Figure 29), following the methodology proposed by Reilly [41] for the morphological study of historic photographs. In the works where it was possible, the masked peripheral margins made equivalent to a pure Dmin area, that is, without any trace of greasy ink, were also analysed.

Using the desktop equipment, a minimum of nine measurement points were set for each work: three measurements in Dmax areas, three in Dmid areas, and three in Dmin areas.

Additionally, whenever possible, three measurements were taken on the reverse side of the primary support, as well as other possible areas of interest (retouches, deteriorated areas, etc.). In the case of the mobile equipment, the number of points per area was reduced, but in all cases, a minimum of one point was measured for each representative area of the image (Dmin, Dmid, and Dmax), along with the reverse of the primary support.

The same methodology was used for the optical microscopy observation of the bromoils. A USB digital surface microscope, Hot^®^ HT—60L, with Hotviewer^®^ v2.7.9 software was used to carry out inspections at 80× and 180×. A revision of the works at 100× was also carried out with an Olympus^®^ BX51 optical microscope coupled to a camera, Optika^®^ 4083.B0.5, to take images using the Optika^®^ Vision Lite programme.

The system for securing the works during measurement had to be adapted to the geometric specifics of each device used. For example, for the desktop equipment, originally designed for analysing small samples, a precision lift platform system with conservation cardboard support adapted to the device was developed to allow horizontal securing of the works, ensuring stability and avoiding vibrations, as the original stage of the desktop spectrometer was insufficient to optimally support the works. Glass weights were added around the analysis area to prevent any interference from vibrations of the sample during measurement. In the case of the mobile equipment, the securing was done vertically using an easel that held a Plexiglas^®^ sheet, where the work was fixed with Melinex^®^ mounting corners and strips.

## 4. Conclusions

In this study, 16 bromoil prints were analysed using FTIR spectroscopy in reflection mode. The selected bromoil samples cover a broad temporal range (~1920–2010) and feature a diverse profile of authors. This includes a heterogeneous spectrum of possible bromoil typologies with eventual variations in the materials used and differences in the fabrication processes, making it plausible to represent diverse compositions and structures through characterisation. This study was conducted using two FTIR instruments, both in external reflection mode: a desktop device and a mobile one.

The results demonstrate that FTIR spectroscopy in external reflection mode is a valuable technique for the study and characterisation of bromoil prints, both in terms of the results it provides and its status as a non-destructive and non-invasive technique. We can confirm that the methodology used for the study of bromoil samples has allowed us to clearly detect the compounds present and make attributions about the strata to which they belong. Cellulose has been described as the major component of the paper support; we have been able to identify the barite layer when this stratum is present in the work immediately above the paper and recognise gelatine and discern its function from the usual photographic colloids where the image-forming material is suspended in it, and the oils and some pigments have been detected in the most superficial layer as image-forming materials (Prussian blue and iron oxides). Regarding the advantages and limitations of the equipment used for this study, we can assert that desktop instrumentation usually provides better spectrum resolution, especially for the identification of oils and their degree of polymerisation, but the mobile instrumentation, which generally covers a broader spectral range, has been decisive in detecting barium sulphate, the main component of the barite layer.

This study shows that the characteristic compounds of bromoil prints that can be identified using external reflection FTIR spectroscopy are the drying oil and some iron-based pigments of the greasy ink of the image and the gelatine layer, onto which the ink is placed. It is demonstrated that, for these to be reliably attributed to the main layers of a bromoil positive and, therefore, be key identifiers of this photographic process, the image-forming materials (oil and pigments) must be detected with a response intensity directly proportional to the image density. Additionally, the gelatine layer should show a response inversely proportional to the image density in terms of band definition and, in some cases, relative intensity, as it is located beneath the ink stratum.

This article resolves some knowledge gaps and contradictions present in the specialised literature: it reveals that it is possible to find bromoils without a barite layer and refutes the idea that this stratum is a key identifier of bromoils, a notion widely accepted in the previous literature. Furthermore, following our study, we propose reconsidering the use of the term colloid or emulsion in bromoil copies to refer to the gelatine layer and suggest understanding it more as a matrix or intermediary stratum intended to retain the image ink. After a deep exploration of the phenomena that occur during the process and production of bromoil prints, the first model for representing the structure of bromoil copies based on evidence from instrumental FTIR spectroscopy analysis is proposed. From these results, it is suggested that bromoils can be described as processes of three or four layers, depending on the presence or absence of the barite layer in each specific case.

No significant variations in the main compounds have been found according to author, context, and/or era. Variations have only been found attributable to the degree of oxidation and polymerisation of the oils, in accordance with the age of the copies, but are not decisive for the identification of the process.

## Figures and Tables

**Table 1:** List of selected bromoil works as objects of study.

Historic Bromoil Photographic Samples			
Sample ID	Author	Approximate Date	Collection
*Cortina_001*	Josep Cortina Molist	Late 1940s to approx. 1950s	Josep Maria Cortina private collection
*Cortina_002*	″	″	″
*Cortina_003*	″	″	″
*Cortina_004*	″	″	″
*Cortina_005*	″	″	″
*Janini_018*	Joaquim Pla Janini	Undated (probably 1930s or earlier)	Vives Pla Family private collection
*Janini_020*	″	″	″
*Janini_021*	″	Undated (between 1920 and 1960)	″
*Janini_026*	″	Undated (between 1920 and 1960)	″
*Janini_028*	″	Undated (probably 1936 or earlier)	″
*Janini_030*	″	Undated (probably 1931 or earlier)	″
*Janini_039*	″	Undated (probably early 1940s or later)	″
*Janini_050*	″	Undated (probably 1935 or earlier)	″
*Janini_051*	″	Undated	″
*Balanya_001*	Jaume Balanyà	2009–2010	Jaume Balanyà private collection
*Balanya_002*	″	″	″
*Balanya_003*	″	″	″

**Table 2:** Images of the selected bromoil works as objects of study.

* 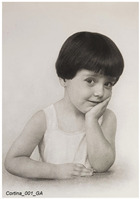 *	* 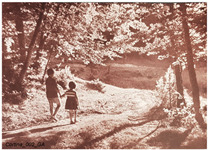 *	* 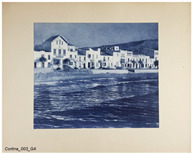 *
* 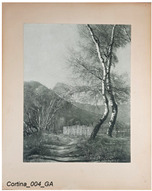 *	* 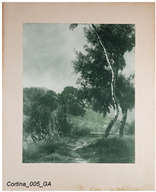 *	* 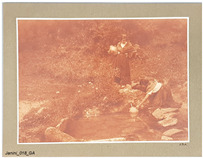 *
* 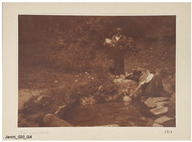 *	* 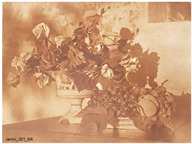 *	* 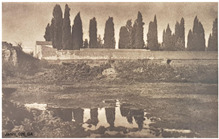 *
* 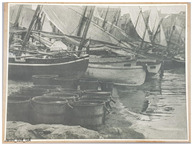 *	* 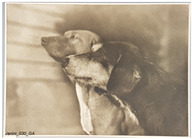 *	* 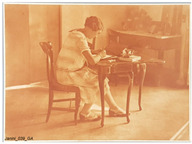 *
* 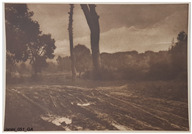 *	* 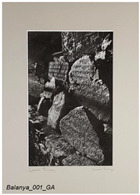 *	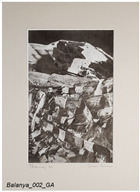
* 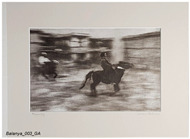 *		

## Data Availability

Raw data were generated at Scientific and facilities of the Research Group of Conservation of Cultural Heritage, University of Barcelona. Derived data supporting the findings of this study are available from the corresponding author on request.
